# Effects of an abnormal mini-mental state examination score on postoperative outcomes in geriatric surgical patients: a meta-analysis

**DOI:** 10.1186/s12871-019-0735-5

**Published:** 2019-05-15

**Authors:** Shuang-jiao Cao, Dongxu- Chen, Lei Yang, Tao Zhu

**Affiliations:** Department of Anesthesiology, West China Hospital, Sichuan University, Chengdu, 610041 People’s Republic of China

**Keywords:** MMSE, Geriatric, Outcomes, Postoperative delirium, Mortality, Meta-analysis

## Abstract

**Background:**

Perioperative cognitive impairment (CI) following surgeries is prevalent in geriatric surgical population aged 60 and older. This meta-analysis was designed to investigate whether the Mini-Mental State Examination (MMSE) has prognostic value on adverse outcomes in aged surgical patients.

**Methods:**

PubMed, Cochrane, Embase and Medline through the Ovid were searched. Meta-analyses were carried out for CI versus non-cognitive impairment (NCI). Quality of evidence was assessed by the GRADE approach.

**Results:**

One randomized controlled trial, two retrospective cohort trials, and 18 prospective cohort trials were included in the meta-analysis. Perioperative diagnosis of CI by the MMSE had higher rates of patients suffering from postoperative delirium (POD) [odd ratio (OR) 5.02, 95% confidence interval (CI) 3.27, 7.71, *P* < 0.00001], in-hospital mortality (OR 7.51, 95% CI 2.17, 26.02, *P* = 0.001), mortality within 1 year (OR 2.53, 95% CI 1.95,3.29, P < 0.00001). Postoperative CI patients had no extended length of stay in orthopedic [standardized mean difference (SMD) -0.10, 95% CI -0.20, 0.17, *P* = 0.91)] nor rehabilitation wards ((SMD, 0.04; 95% CI, − 0.23 to 0.31; *P* = 0.78).

**Conclusion:**

Older patients with perioperative CI were more likely to suffer from POD and mortality. The MMSE showed certain value on risk stratification and prognosis evaluation in geriatric surgical population.

**Trial registration:**

PROSPERO CRD42018108739.

**Electronic supplementary material:**

The online version of this article (10.1186/s12871-019-0735-5) contains supplementary material, which is available to authorized users.

## Backgrounds

Surgical safety and success rates have improved considerably as a result of remarkable medical breakthroughs, which has led to the extension of life expectancy and the rise in the number of aged patients undergoing surgical procedures [1]. Compared to 2017, the number of persons aged 60 and older is expected to more than double by 2050 and more than triple by 2100, thereby rising from 962 million globally in 2017 to 2.1 billion in 2050 and 3.1 billion in 2100 [2], which will necessitate an increasing demand for operations in aged individuals. In England, less than 1.5 million people over 75 years old underwent surgery between 2006 and 2007, contrasted with 2.5 million between 2014 and 2015. Among these 2.5 million patients, up to 30% were above 85 years of age. Similarly, in Australia, female patients aged 85 years and older have tended to be the largest group of the overall emergency surgical population [3]. In America, a research [4] study reported that in 2015, patients 65 years of age and older accounted for 34.1% of all surgeries.

Comorbidities are more common in older patients, the ability for their bodies to compensate decreases, and some health problems may be underdiagnosed; these factors contribute to an inability to tolerate surgery [5]. Surgery in older patients presents medical workers with formidable challenges as they must weigh long-term benefits and risks carefully to make the best surgical decisions. Previous studies have reported that both preexisting and new-onset cognitive impairment (CI) following surgeries, which has been observed in 19–83% of elderly patients varying with age and type of surgery [6, 7], was associated with an increased incidence of postoperative complications such as delirium. Other long-term problems and poor outcomes have also been of concern, such as mortality, impaired functional status, readmission, prolonged hospitalizations and increased expenses [3, 8–10]. The Mini-Mental State Examination (MMSE) is long established and the most widely used instrument to help clinicians detect cognitive impairment and grade the severity of cognitive change [11]. This tool is frequently applied in the perioperative period of older patients due to its adequate performance in a rule-out capacity [12]. However, several studies have confirmed that the MMSE score could be a predictor of adverse postoperative outcomes, but those findings are limited by the sample sizes or other confounders. Thus, whether the MMSE could be an indicator of the prognosis of geriatric surgical patients remains unclear. To our knowledge, there have been no reviews systematically exploring and quantifying the association between impaired cognition diagnosed by the MMSE and different postoperative outcomes.

We conducted this systematic review to investigate whether the MMSE used as a perioperative assessment tool has prognostic value on adverse clinical outcomes in aged surgical patients and to search for a better understanding and guidance for clinicians to evaluate and make optimal patient care decisions.

## Methods

### Data sources and search strategy

This systematic review was performed and presented following the principles of the Preferred Reporting Items for Systematic Reviews and Meta-Analyses (PRISMA) statement [13] and Assessing the Methodological Quality of Systematic Reviews (AMSTAR) guidelines. The protocol was registered with the International Prospective Register of Systematic Reviews (http://www.crd.york.ac.uk/PROSPERO/display_record.php?ID=CRD42018108739 RecordID = 108,739). A systematic literature search was conducted for studies published from 1995 to April 2018 by searching PubMed, the Cochrane Library, Embase and Medline through Ovid. The search terms combined medical subject headings and keywords related to the MMSE, surgery and outcomes. The sensitive search was performed by using the following terms: “cognition”, “cognitive”, “delirium”, “complication”, “outcome”, “length of stay”, “surgery”, “operation”, “operative”, “procedure”, “Mini-Mental State Examination”, “MMSE”. The literature search strategy is provided in Additional file 1: Material 1.

### Study selection and data collection

This systematic review and meta-analysis included randomized controlled trials (RCTs), prospective cohort studies, and retrospective cohort studies. Eligible studies were included if they met the following criteria:

1. Surgical patients aged 60 years and older.
2. The MMSE was applied during the preoperative or postoperative period or at admission to the rehabilitation ward/hospital/facility.
3. The outcomes of interest included all postoperative complications, especially postoperative delirium (POD), hospitalization days, mortality (in-hospital mortality and long-term mortality).
4. Quantitative data were reported to compare each MMSE group with outcomes.

The initial step was based on screening titles and abstracts to exclude irrelevant studies. Second, the full contents of potentially eligible studies were read. Additionally, data were extracted and collected. The extracted characteristics of the studies included author, published year, study design, type of surgery, sample size, patient age, initial time of MMSE assessment (before or after surgery), the cutoff point of the MMSE scores to define cognitive impairment, and reported outcomes along with their definitions and follow-up duration. Outcomes that had been observed in the same way in more than two studies were included in the meta-analysis. The number of events and the number of participants in each group were extracted for dichotomous outcomes. Mean, standard deviation and the number of participants were extracted for continuous outcomes. The screening and extraction were conducted by two authors separately. Discrepancies with regard to eligibility were determined by a third author.

### Statistical analysis

We used Review Manager (RevMan, version 5.3 for Windows, Oxford, UK; The Cochrane Collaboration, 2008) to perform the meta-analysis, which included generating forest plots and testing for heterogeneity and overall effects. Dichotomous outcomes were analyzed by the Mantel–Haenszel method and odds ratios (ORs), while continuous outcomes were analyzed by the inverse variance method and standard mean difference (SMD). Random effects models were used for all analyses. Heterogeneity was assessed using the I^2^ statistic. For this measure, 0 to 50%, 50 to 75%, and 75 to 100% represented low, moderate, and high levels of heterogeneity, respectively [14]. Moderate to high levels of heterogeneity (I^2^ > 50%) between studies were investigated by several subgroup analyses including preoperative and postoperative MMSE, length of stay in orthopedic wards and rehabilitation wards, in-hospital mortality and 1-year mortality. Sensitivity analyses were conducted to explore the impact of imputing nonsignificant results on pooled effects. *P* values of less than 0.05 indicated statistical significance. Funnel plots were generated in Review Manager, and Egger’s test was performed in STATA15.0 (StataCorp LLC, Texas) to assess publication bias [15].

### Quality assessment and risk of bias

Two authors assessed the risk of bias independently. Disagreement was resolved by consulting other authors. We used the Newcastle Ottawa Quality Assessment Scale [16] (NOS) for Cohort Studies (range of 0 to 9 stars), a tool for the critical appraisal of eligible cohort studies. We regarded a study that scored seven or more stars as high quality and five or less stars as poor quality. We used the Cochrane Risk of Bias tool, which allows an assessment of low, moderate, or high risk of bias [17] to analyze the quality of RCTs.

We used the GRADE (Grading of Recommendations, Assessment, Development, and Evaluation) [18] approach to rate the quality of the evidence for postoperative delirium, length of stay, readmission to hospital and admission to nursing home within 1 year. Evidence was judged as high, moderate, low and very low in consideration of risk of bias, inconsistency, indirectness, imprecision and other considerations. We used the GRADEpro GDT to generate the evidence profile.

## Results

**Fig. 1:**
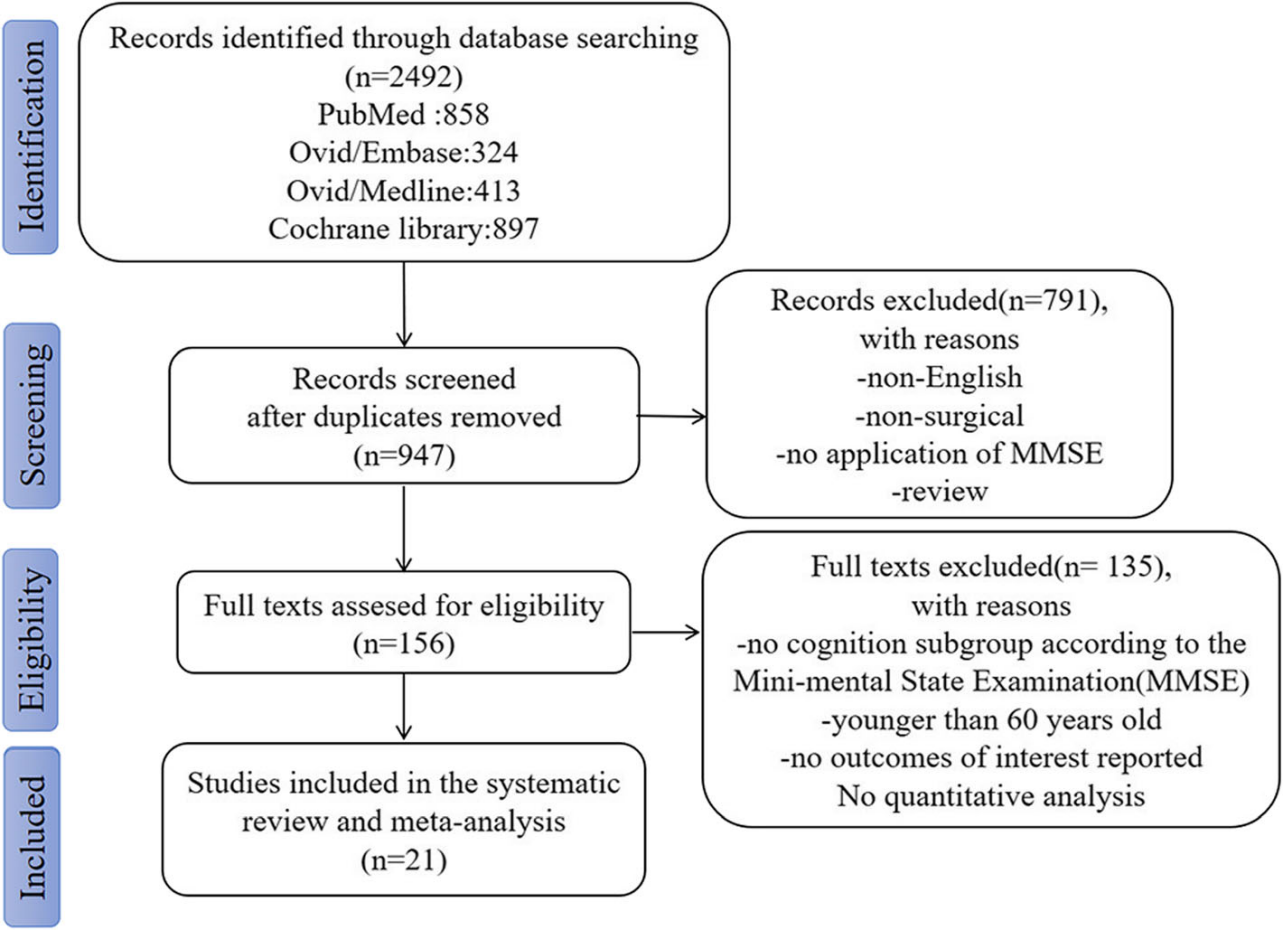
PRISMA flow chart of study selection

We identified a total of 2492 records. After removal of duplicates, we screened 947 titles and abstracts, of which 156 full text articles were selected for eligibility. Twenty-one studies met the inclusion criteria for the systematic review (Fig. 1).

The designs of the included studies were a randomized controlled trial [19](*N* = 1), retrospective cohort trials [7, 20](*N* = 2), and prospective cohort trials [21–38](*N* = 18). Two sets of data were extracted from the intervention group and control group of the RCT (Table 1). When defining cognitive impairment or cognitive intact, most of the included studies used the MMSE cutoff score of 24, while another 6 studies used a cutoff score of 18 [31], 20 [22], 26 [25], 27 [27, 32], or 28 [36]. Considering the inconsistency of the cutoff point, we grouped our study populations into CI and NCI based on a clear definition by every study instead of selecting a specific cutoff point.

**Table 1:** Study characteristics. MMSE, the Mini-Mental State Examination; POD, postoperative delirium; RCT, randomized controlled trials

Study	Design	Surgery type	Sample size	Participant	initiation of MMSE	Cutoff point of MMSE	Reported outcomes of interest
Beloosesky 2002 [26]	Prospective cohort study	Hip surgery	153	≥65 yr	Before surgery	24	In-hospital mortality
Bliemel 2015 [27]	Prospective cohort study	Hip surgery	399	≥60 yr	Before surgery	27	1-year mortality
Brouquet 2010 [25]	Prospective cohort study	Abdominal surgery	118	≥75 yr	Before surgery	26	POD
Guo 2014 [29]	Prospective cohort study	Hip surgery	244	>60 yr	Before surgery	24	Total hospitalization days (surgery and rehabilitation)
							1-month/6-months/1- year mortality
Häkkinen 2007 [28]	Prospective cohort study	Hip surgery	117	≥65 yr	After surgery	24	Length of stay in orthopedic/rehabilitation ward
							1-year mortality
Huusko 2000 [19]	RCT	Hip surgery	243	≥65 yr	After surgery	24	3 month/1 year mortality
							Length of stay in rehabilitation ward
Jones 2017 [31]	Prospective cohort study	Hip surgery	383	≥65 yr	After surgery	18	6 months Mortality
							Length of stay in orthopedic wards
Kalisvaart 2006 [30]	Prospective cohort study	Hip surgery	603	≥70 yr	Before surgery	24	POD
Karni 2013 [33]	Prospective cohort study	Hip surgery	60	≥65 yr. Female	After surgery	24	Length of stay in rehabilitation ward
Kratz 2015 [32]	Prospective cohort study	general, abdominal, and trauma surgery	178	>70 yr	Before surgery	27	POD
Lee 2016 [7]	Retrospective cohort study	lumbar spine surgery	129	>65 yr	Before surgery	24	POD
							Length of stay in hospital
Moncada 2005 [35]	Prospective cohort study	Hip surgery	48	≥65 yr	After surgery	24	Length of stay in orthopedic/rehabilitation ward
							POD
Morghen 2011 [34]	Prospective cohort study	Hip surgery	386	≥65 yr	After surgery	24	Length of stay in rehabilitation ward
Osse 2012 [36]	Prospective cohort study	Cardiac surgery	125	≥70 yr	Before surgery	28	POD
Otano 2015 [38]	Prospective cohort study	Hip surgery	285	≥65 yr	After surgery	24	In-hospital mortality Length of stay in rehabilitation ward
Reissmüller 2006 [37]	Prospective cohort study	Cardiac surgery	107	≥60 yr	Before surgery	24	POD
Rolland 2004 [22]	Prospective cohort study	Hip surgery	61	≥70 yr	After surgery	20	Length of stay in rehabilitation ward
Ruggiero 2016 [21]	Prospective cohort study	Hip surgery	514	≥65 yr	After surgery	24	1-year mortality
Schaller2012 [23]	Prospective cohort study	Hip surgery	173	≥65 yr	After surgery	24	1 year mortality
Witlox 2009 [24]	Prospective cohort study	Hip surgery	76	≥75 yr	Before surgery	24	POD
Yukako 2016 [20]	Retrospective cohort study	Colorectal surgery	156	≥75 yr	Before surgery	24	POD

**Fig. 2:**
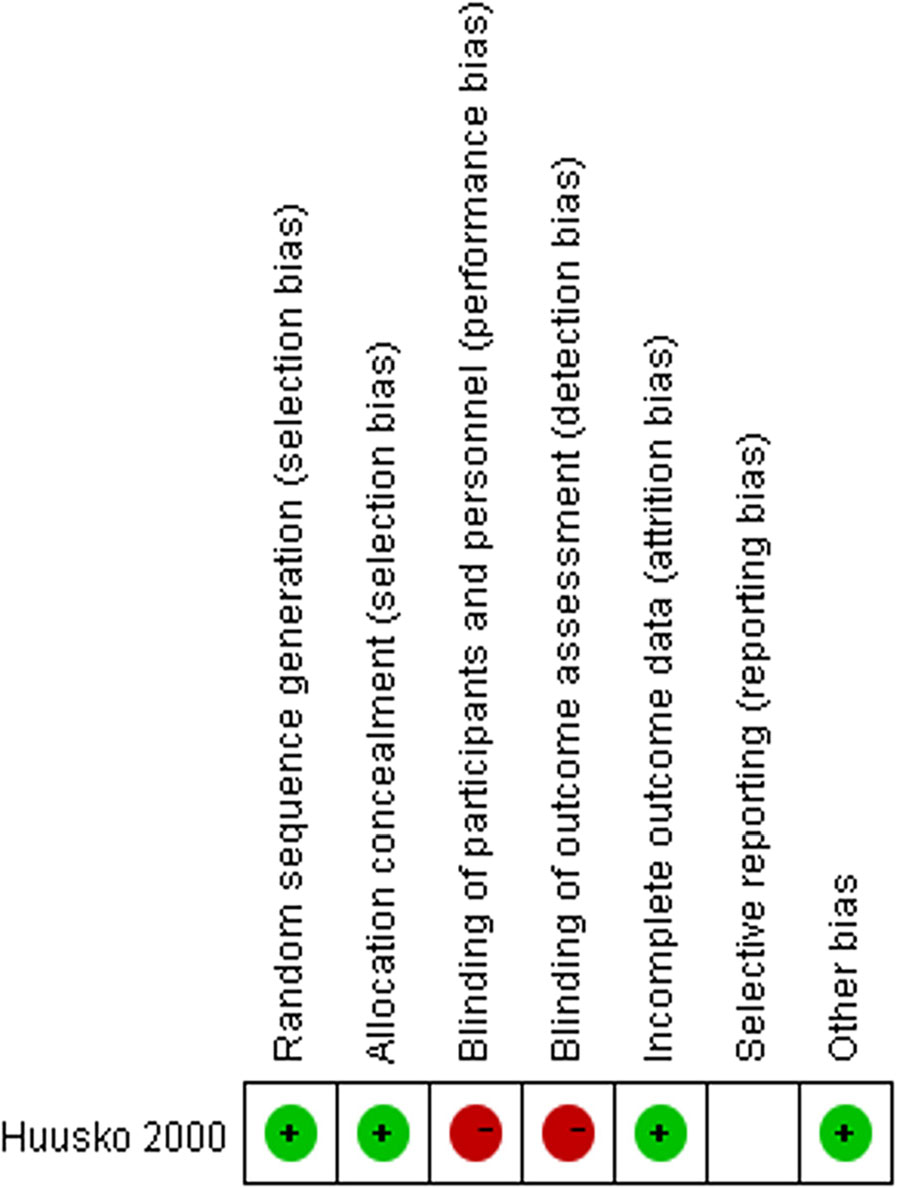
Risk of bias for 1 Randomized controlled trail

Generally, most of the included studies were judged to be of moderate to high quality. The risk of bias concerns in all the cohort studies were frequently about the comparability of the CI and NCI groups. Other risks of bias included short follow-up durations and the presence of outcomes at the start of the studies. The RCT was at high risk of performance and detection bias and unclear risk of reporting bias (Table 2, Fig. 2).

**Table 2:** Risk of bias for cohort trails

Study	Selection				Comparability	outcome			Score
	Represent-ativeness of the exposed cohort	Selection of the non-exposed cohort	Ascertainment of exposure	Demonstration that outcome of interest was not present at start of study	Comparabili-ty of Cohorts on the Basis of the Design or Analysis	Assess-ment of outco-me	Was follow-up long enough for outcomes to occur	Adequacy of follow -up of cohorts	
Beloosesky 2002 [26]	★	★	★	★		★		★	6
Bliemel 2015 [27]	★	★	★	★	★	★	★	★	8
Brouquet 2010 [25]	★	★	★	★		★	★	★	7
Guo 2014 [29]	★	★	★	★	★	★	★	★	8
Häkkinen 2007 [28]	★	★	★	★	★	★	★	★	8
Huusko 2000 [19]	★	★	★	★	★	★	★	★	8
Jones 2017 [31]	★	★	★	★		★	★	★	7
Kalisvaart 2006 [30]		★	★	★	★	★		★	6
Karni 2013 [33]	★	★	★	★		★	★	★	7
Kratz 2015 [32]	★	★	★		★		★	★	6
Lee 2016 [7]	★	★	★	★		★	★	★	7
Moncada 2005 [35]	★	★	★	★	★	★		★	7
Morghen 2011 [34]	★	★	★	★	★	★	★	★	8
Osse 2012 [36]	★	★	★	★		★	★	★	7
Otano 2015 [38]	★	★	★	★	★	★	★	★	8
Reissmüller 2006 [37]	★	★	★	★		★	★	★	7
Rolland 2004 [22]	★	★	★	★	★	★	★	★	8
Ruggiero 2016 [21]	★	★	★	★		★	★	★	7
Schaller2012 [23]	★	★	★	★	★	★	★	★	8
Witlox 2009 [24]	★	★	★	★		★	★	★	7
Yukako 2016 [20]	★	★	★			★	★	★	6

### Postoperative delirium (POD)

**Fig. 3:**
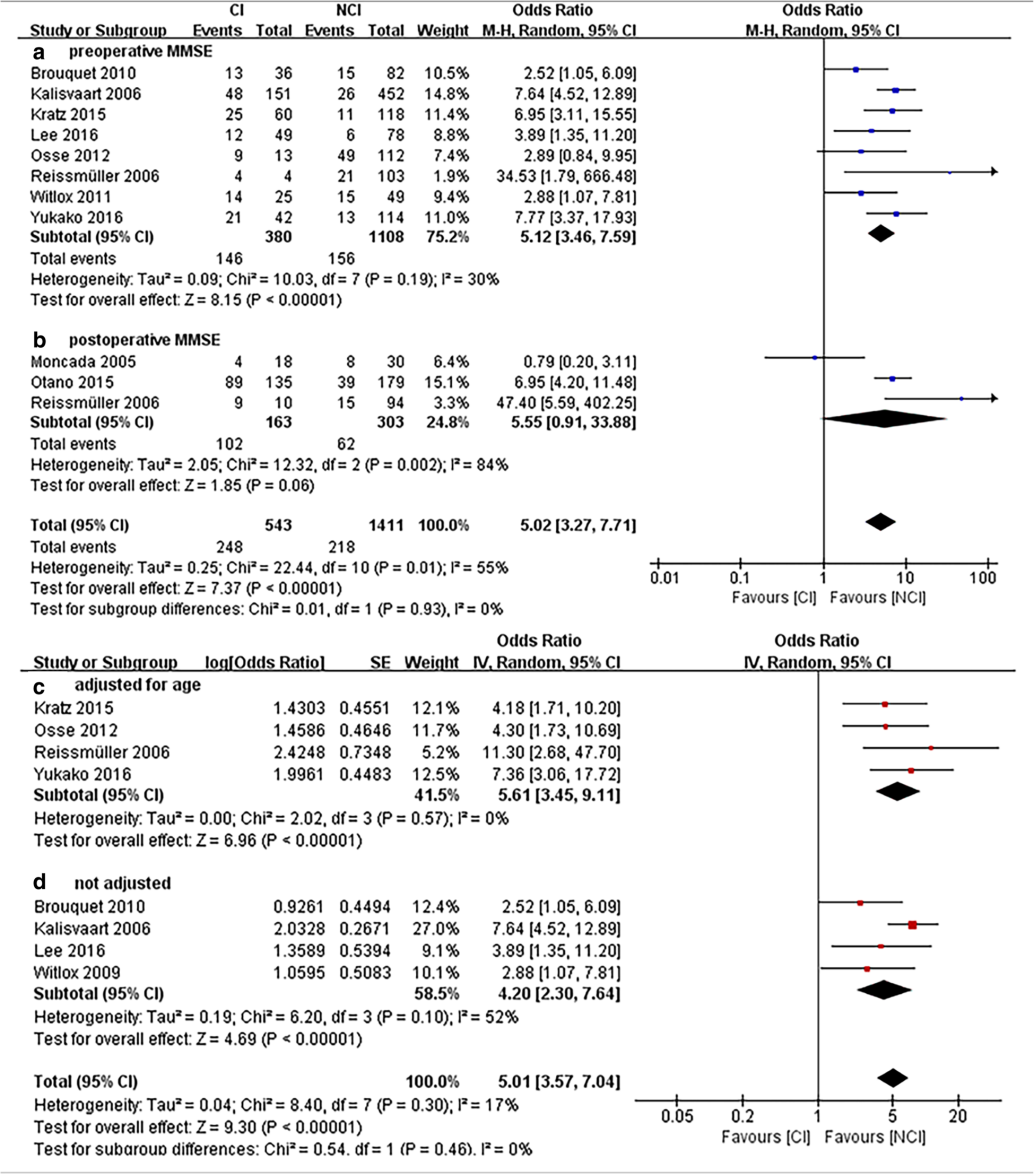
Forest Plot of postoperative delirium (POD). **a**, preoperative MMSE; **b** postoperative MMSE; **c** preoperative MMSE adjusted for age; **d**, preoperative MMSE not adjusted for age

Ten studies [7, 20, 24, 25, 30, 32, 35–38] reported POD including 1411 patients with NCI and 543 with CI (Fig. 3). Patients with perioperative CI had a higher rate of POD compared with NCI patients [odds ratio (OR), 5.02; 95% confidence interval (CI), 3.27 to 7.71; *P* < 0.00001] (Fig. 3).

In a subgroup analysis, three studies [35, 37, 38] used the MMSE postoperatively, while eight [7, 20, 24, 25, 30, 32, 36, 37] studies used the MMSE preoperatively. One study [37] applied the MMSE both preoperatively and postoperatively. The rate of POD was higher in the preoperatively diagnosed CI group than in the preoperatively diagnosed NCI group (OR, 5.12; 95% CI, 3.46 to 7.59; P < 0.00001; I^2^ = 30%) (Fig. 3a). Postoperatively diagnosed CI did not increase the rate of POD (OR, 5.55; 95% CI, 0.91 to 33.88; *P* = 0.06; I^2^ = 84%), and substantial heterogeneity existed (I^2^ = 84%) (Fig. 3b), then we conducted a sensitivity analysis to explore the stability of the latter results. In the subgroup of postoperative MMSE for the outcome of postoperative delirium, we found that the postoperative CI group showed a higher rate (OR, 0.07; 95% CI, 0.01 to 0.45; *P* = 0.006; I^2^ = 66%) after excluding the study “Moncada 2005” [35], and when the risk ratio (RR) or risk difference (RD) was calculated, the rates were higher (Table 3).

**Table 3:** Sensitivity analysis of postoperative delirium in the postoperative subgroup. Figures are Mantel-Hanzel point estimates

comparison	Point estimate(95% CI)	P	I^2^
Primary analysis	OR 5.55(0.91, 33.88)	0.06	84%
Sensitivity analysis			
Exclude the study “Moncada 2005” [35]	OR 13.56(2.24, 81.97)	0.005	66%
Alter effect measure: Relative risk	RR 2.78(1.27, 6.05)	0.01	84%
Alter effect measure: Relative difference	RD 0.39(0.04, 0.74)	0.03	92%

We carried out another subgroup analysis by whether the eight studies using MMSE preoperatively adjusted for age to decrease the effects of age on postoperative delirium (Fig. 3c, d). After adjusting for age, the rate of POD was higher in the preoperatively diagnosed CI group than in the preoperatively diagnosed NCI group (OR, 5.61; 95% CI, 3.45 to 9.11).

### Length of stay in hospitals

**Fig. 4:**
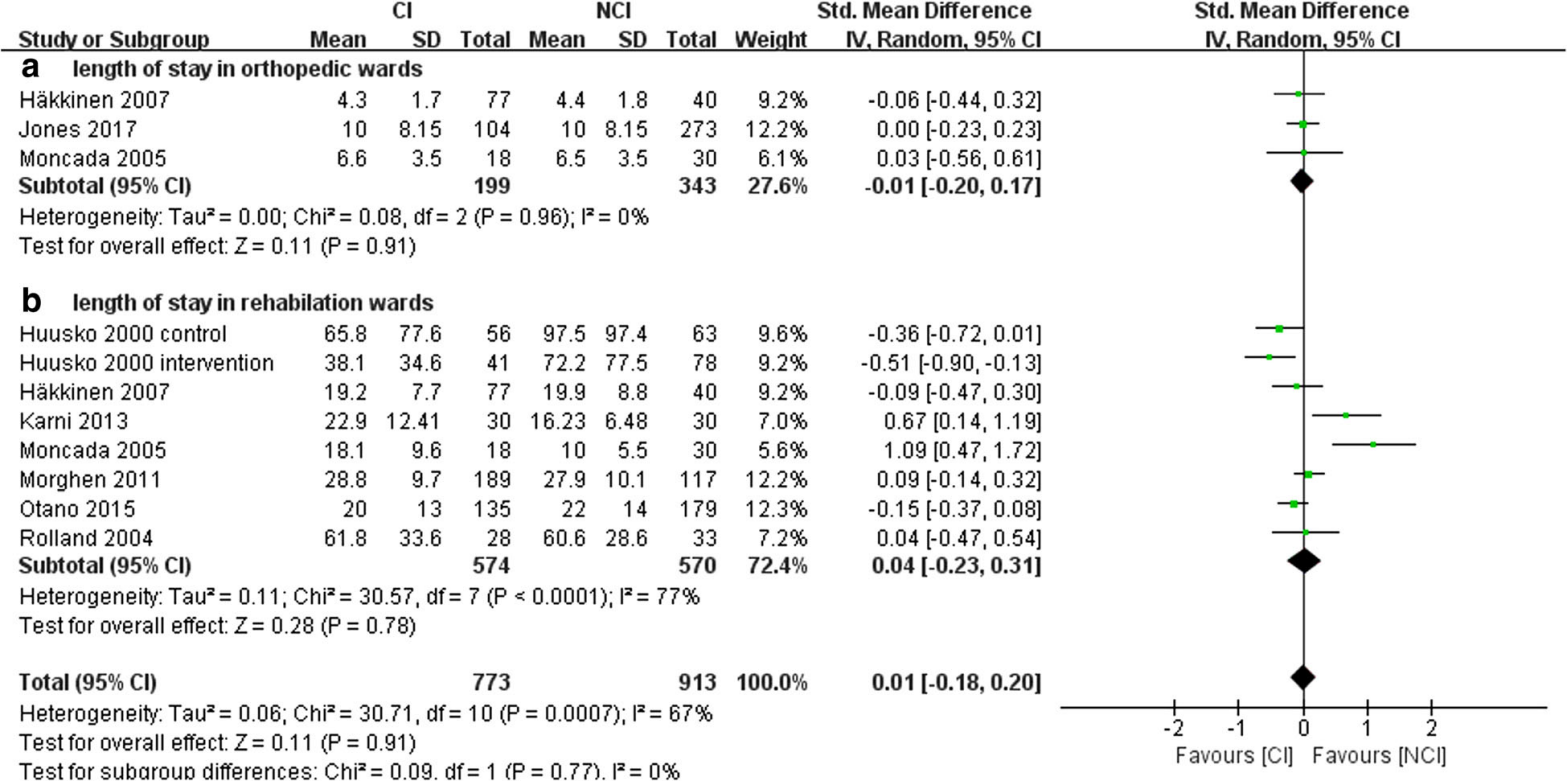
Forest Plot of postoperative MMSE and length of stay in hospitals. **a**, orthopedic wards; **b** rehabilitation wards

We included 8 studies [19, 22, 28, 31, 33–35, 38] consisting of 913 patients with NCI and 773 with CI for meta-analysis of postoperative MMSE and length of stay in hospital. CI did not increase length of stay (SMD, 0.01; 95% CI, − 0.18 to 0.20; *P* = 0.91) (Fig. 4). Three of the trials [28, 31, 35] reported length of stay in orthopedic wards or in the acute perioperative phase. Seven trials [19, 22, 28, 33–35, 38] reported stay length in geriatric wards or rehabilitation wards after the acute phase. We then conducted a subgroup analysis and found CI did not increase length of stay in in orthopedic wards (SMD, − 0.10; 95% CI, − 0.20 to 0.17; P = 0.91) nor in rehabilitation wards (SMD, 0.04; 95% CI, − 0.23 to 0.31; *P* = 0.78) (Fig. 4a, b).

Two studies [7, 29], using the MMSE preoperatively, defined length of stay in a confusing and inconsistent manner and could not be combined in the meta-analysis.

### Mortality

**Fig. 5:**
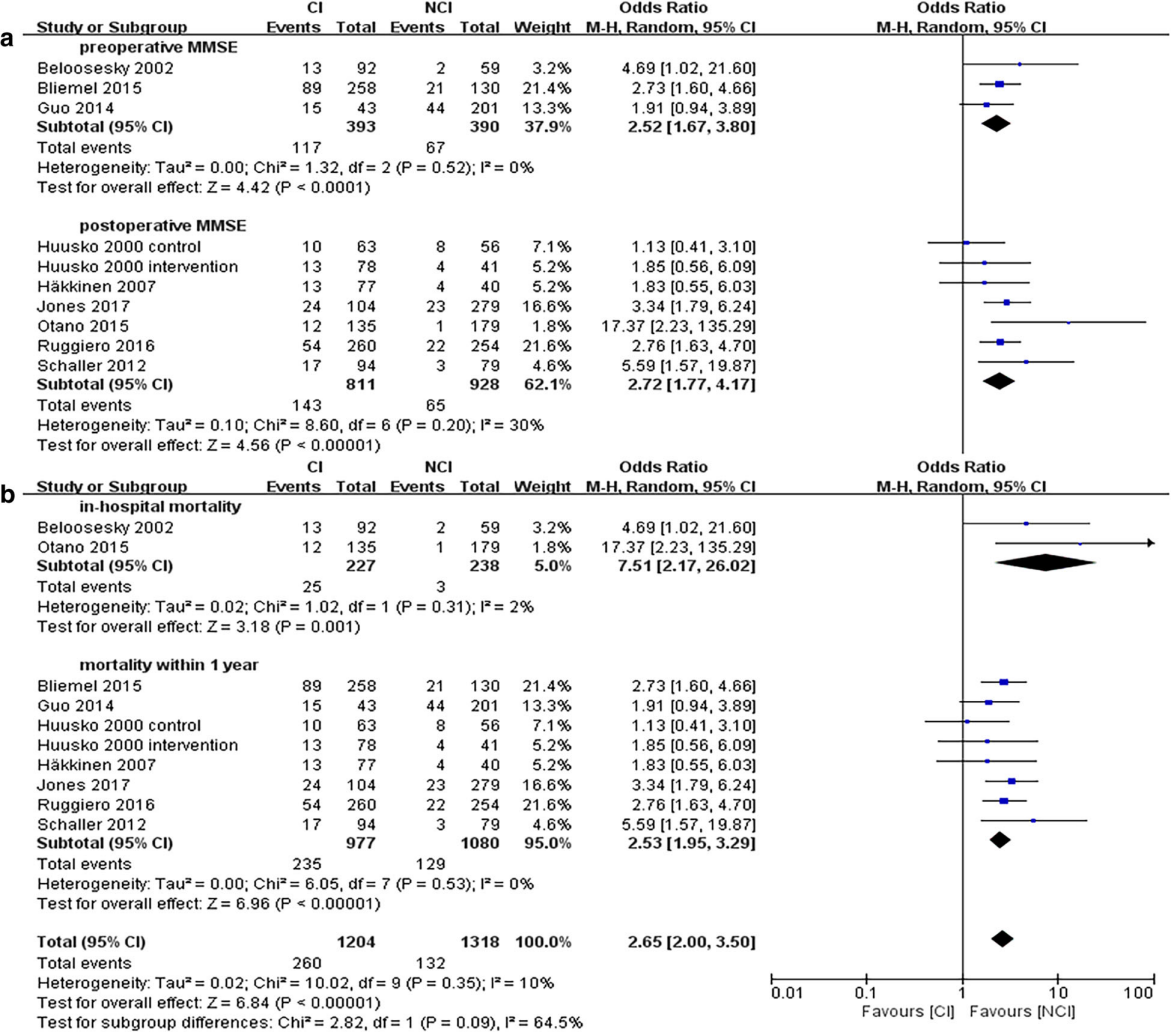
Forest Plot of mortality. **a**, subgroup analysis of preoperative and postoperative MMSE; **b**, subgroup analysis of in hospital mortality and mortality within 1 year

Nine studies [19, 21, 23, 26–29, 31, 38] reported mortality including 1318 patients with NCI and 1204 with CI (Fig. 5). Patients with CI had a higher rate in mortality compared to those with NCI (OR, 2.65; 95% CI, 2.00 to 3.50; *P* < 0.00001). Heterogeneity between the trials was low (I^2^ = 0%).

In the subgroup analysis, patients with preoperative CI [26, 27, 29] presented an increased rate of death. (OR, 2.52; 95% CI, 1.67 to 3.80; *P* < 0.0001). Similar results were also found in the postoperative CI [19, 21, 23, 28, 31, 38] group (OR, 2.72; 95% CI, 1.77 to 4.17; P < 0.00001) (Fig. 5a). The group with perioperative CI had a higher rate of in-hospital mortality [26, 38] (OR, 7.51; 95% CI, 2.17 to 26.02; *P* = 0.001) and mortality within one year [19, 21, 23, 27–29, 31] (OR, 2.53; 95% CI, 1.95 to 3.29; P < 0.00001;) (Fig. 5b).

### Publication bias and quality of evidence

**Fig. 6:**
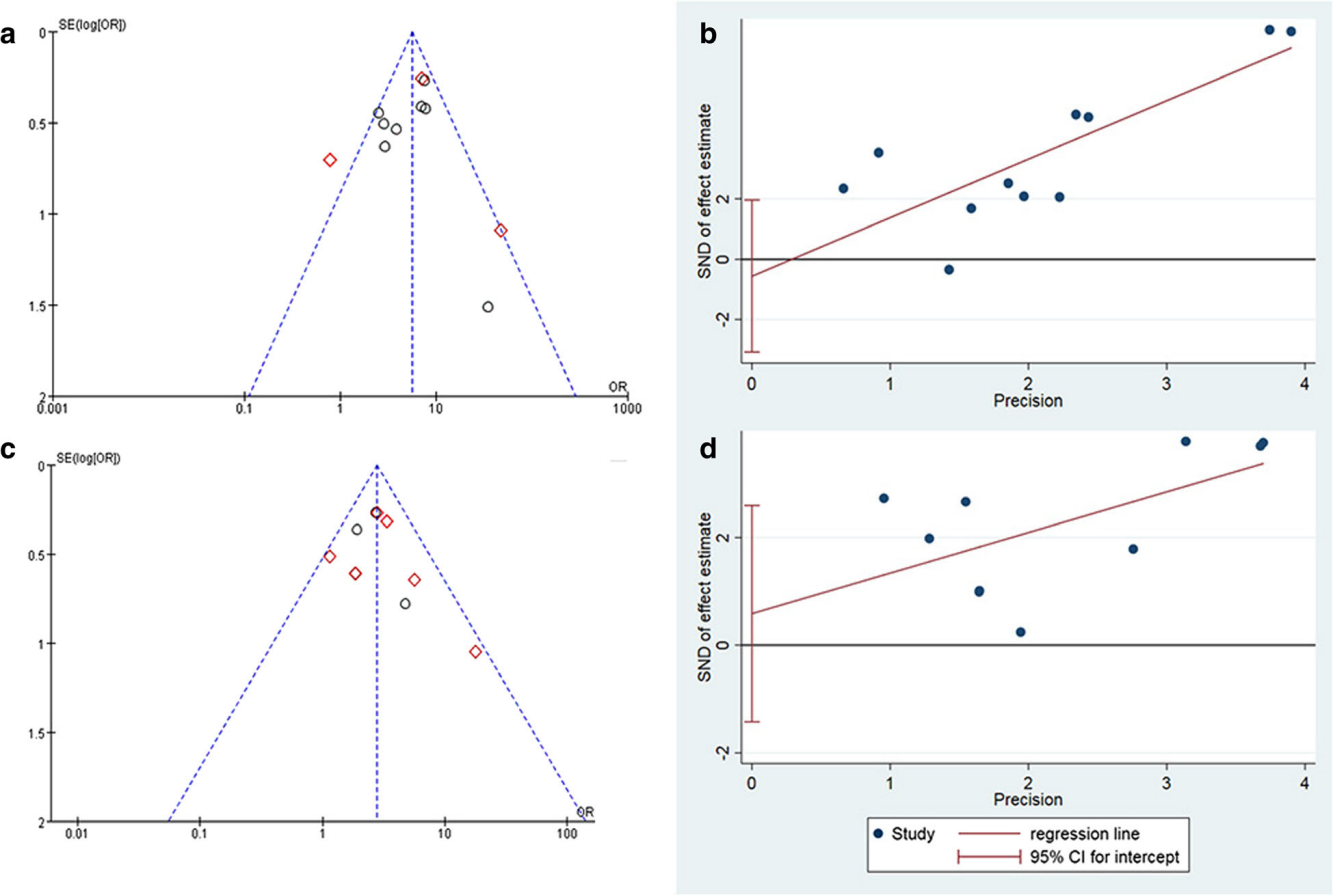
**a**, funnel plot of POD; **b**, egger graph of POD; **c**, funnel plot of mortality; **d**, egger graph of mortality

Quantitative synthesis of POD and mortality involved 11 and 10 sets of data, respectively; thus, we generated funnel plots. To exclude the existence of publication bias by visual inspection, we conducted the Egger test and found there was no evidence of publication bias for the outcomes of POD (*p* = 0.626) and mortality (*p* = 0.520) (Fig. 6).

Based on the GRADE approach, we found a high quality of evidence for POD in the preoperative subgroup. We found a moderate quality of evidence for POD in the postoperative subgroup and length of stay in orthopedic wards (Table 4). We did not rate the quality of evidence for mortality as we included one RCT and nine cohort studies in this outcome.

**Table 4:** GRADE evidence profile for cognitive impaired (CI) patients versus non-CI patients. Explanations: a. asymmetrical funnel plot, b. I^2^ = 84%, c. OR 5.55(0.91, 33.88), d. I^2^ = 74%

Certainty assessment							№ of patients		Effect		Certainty	Importance
№ of studies	Study design	Risk of bias	Inconsistency	Indirectness	Imprecision	Other considerations	CI	NCI	Relative (95% CI)	Absolute (95% CI)		
POD - preoperative MMSE												
8	observational studies	not serious	not serious	not serious	not serious	publication bias strongly suspected very strong association all plausible residual confounding would reduce the demonstrated effect ^a^	146/380 (38.4%)	156/1108 (14.1%)	OR 5.12 (3.46 to 7.59)	315 more per 1000 (from 221 more to 414 more)	⨁⨁⨁⨁ HIGH	CRITICAL
POD - postoperative MMSE												
3	observational studies	not serious	serious ^b^	not serious	serious ^c^	very strong association all plausible residual confounding would reduce the demonstrated effect	102/163 (62.6%)	62/303 (20.5%)	OR 5.55 (0.91 to 33.88)	383 more per 1000 (from 15 fewer to 692 more)	⨁⨁⨁◯ MODERATE	CRITICAL
length of stay in orthopedic wards-postoperative MMSE												
3	observational studies	not serious	not serious	not serious	not serious	all plausible residual confounding would reduce the demonstrated effect	199	343	–	SMD 0.01 lower (0.2 lower to 0.17 higher)	⨁⨁⨁◯ MODERATE	IMPORTANT
length of stay in rehabilitation wards -postoperative MMSE												
6	observational studies	not serious	serious ^d^	not serious	not serious	all plausible residual confounding would reduce the demonstrated effect	477	429	–	SMD 0.2 higher (0.1 lower to 0.49 higher)	⨁⨁◯◯ LOW	IMPORTANT

*CI* Confidence interval, *OR* Odds ratio, *SMD* Standardised mean difference

*Explanations*

^a^ asymmetrical funnel plot

^b^I^2^ = 84%

^c^OR 5.55(0.91, 33.88)

^d^I^2^ = 74%

## Discussion

The principal findings of our meta-analysis are that older patients with perioperative diagnoses of cognitive impairment by the MMSE had higher risk of postoperative delirium, in-hospital mortality and mortality within 1 year. We investigated the timing of assessment with the MMSE and the respective effect on adverse outcomes. Preoperative diagnosis of CI appeared to yield a more significant association with postoperative delirium than postoperatively diagnosed CI, however, according to the sensitivity analysis, the wide variance in the observed effect was due to high heterogeneity, primarily due to the inclusion of the study of Moncada’s; therefore, definitive conclusions cannot be drawn. We suspected that as postoperative delirium is most common on the first and third postoperative days [39], the results of the meta-analysis may have been altered if the onset of delirium was earlier than the timing of postoperative diagnosis of CI; thus, preoperative use of the MMSE may be preferable to predict the incidence of postoperative delirium. Postoperative CI patients did not have an extended stay length in orthopedic wards or rehabilitation wards. However, these results raised questions about insufficient sample sizes and heterogeneity in the eligible studies.

Our systematic review and meta-analysis was novel in providing data showing the predictive value of perioperative assessment by the MMSE on postoperative outcomes in older patients. We also took into consideration the timing of assessment of cognition, as preoperative cognitive impairment revealed a chronic aging-related change while postoperative CI usually developed with acute onset and was confounded by surgery, anesthesia, medication and a stress response [40]. We summarized the data from 21 studies and used both the NOS and the Cochrane risk of bias tool to appraise the quality of selected cohort studies and an RCT, respectively. The methodological quality of studies in our review was fair to high. No significant bias of publication was observed in the report of postoperative delirium and mortality. These factors contributed to more powerful evidence than any single study or previous systematic review that failed to conduct a quantitative data synthesis.

However, there are some potential limitations should be considered. One important limitation of our study was the innate defects and the use of inconsistent cutoff scores for the MMSE across studies, which might have impacted the positive diagnosis rate of cognitive impairment. The MMSE has been validated widely around the world; however, it has been suggested that the MMSE does not perform better as a rule-out tool than a definitive diagnostic tool, which means that for those positive on the MMSE, a more detailed evaluation and inspection are required [12]. The MMSE is not the most efficient tool. Typically, it will take 8 min to complete an assessment in NCI individuals while taking 15 min or longer to evaluate CI patients [41]. Previous studies have indicated that the MMSE would be most suitable in specialist settings compared with community setting and primary care settings due to significant intraobserver differences [42]. The MMSE does not perform well enough in patients with mild cognitive impairment and early dementia [43]. Performance can be disrupted by education, age, language, ethnicity and cultural differences [44], thus optimal cutoff values change in different clinical settings. For instance, threshold values of 21, 23 and 24 are suggested in populations with primary school, high school and university education, respectively [12]. Therefore, we decided to group our study population based on a clear definition of CI by every investigator instead of selecting a specific cutoff score.

Another limitation was that some of our included studies lacked clear discharge criteria and detailed rehabilitation interventions, making the outcome of length of stay less convincing. Selection and measurement biases existed as medical and rehabilitation strength varied from different districts and hospitals. Consequently, the results found in our study need to be interpreted with caution.

Most of our included studies have restricted enrollment or matched the two cognitive groups in terms of age, sex, education levels, and surgery types and so on, even though the variables in individual studies were different. There was an inconsistency distribution of age between the two cognitive groups in some studies (Additional file 2: Material 2), and age did have a negative effect on postoperative outcomes (Additional file 3: Material 3). We could reasonably have concluded that age may act as a confounder. Adjustment for a wide range of potential confounders in individual studies were listed in Additional file 4: Material 4. We carried out a subgroup analysis by whether studies adjusted for age to decrease the effects of age on postoperative delirium. we failed to do Subgroup analysis of other outcomes owing to the lack of adjusted ORs in original studies.

Delirium and dementia are among the most common causes of cognitive impairment in clinical settings, yet their interrelationship remains poorly understood and they are often either unrecognized or mistaken for each other [45]. Our study had limitations that warrant consideration. Cognitive status was measured using the general screening tool, MMSE, and did not specifically identify dementia or delirium., Almost all our included studies chose not to use the more specific concepts of delirium or dementia, as they frequently relied on second-hand observations or precise diagnostic methods instead of a single examination like MMSE, and thus were often under- or misdiagnosed, especially by non-psychiatric staffs. Instead, they investigated the broader concept of cognitive impairment using MMSE. Thus we classified the patients into two cognitive groups and thereby minimize its false positive and negative results. We have not investigated whether acute or chronical cognitive impairment had different surgical outcomes as almost all our included studies did not provide baseline level of MMSE or a clear history of pre-existing dementia prior to admission or earlier. As a matter of fact, it was hard and exhausting for clinicians to get these medical history of patient especially geriatric ones in clinical settings. Previous studies proved that both chronic and acute cognitive impairment were independent risk factors for a poorer outcome after hip fracture [46–50]. Yet it still remained unclear which one would be involved with worse surgical outcomes. Beyond all question, dementia as well as delirium during an acute and intense stress, like surgical procedures or hip fracture, unveils the preexisting or subclinical frailty of the geriatric individual. It is critically important for Clinicians and researchers to screen for this part of surgical patients and pay attention for the presence of a frailty syndrome, which make sense of our study and the perioperative application of MMSE. Because of its wide acceptance as a general screening instrument for cognitive dysfunction, and because the test can be performed at the bedside within a relatively short period of time.

Some directions for future research should be drawn out from our findings. With the population aging, the demand for surgery in the elderly is growing. There are striking differences in the tolerance, recovery and clinical outcomes between cognitive impaired and cognitive intact older patients following surgery. The American College of Surgeons has provided both preoperative and postoperative rounding checklists for geriatric surgical patients, including a strong recommendation of the assessment and documentation of cognitive dysfunction [51, 52]. Our review further recommends that the MMSE may be used as a reliable preoperative screening tool as well as a postoperative follow-up index in geriatric surgical settings to optimize risk stratification, assess prognosis in this population, and provide indications for early and effective interventions. Moreover, further research is needed to search for more and better assessment instruments to help make clinical decisions for older patients.

## Conclusions

We found that older patients with perioperative CI were more likely to suffer from postoperative delirium, in-hospital mortality and mortality within 1 year. The MMSE showed certain value on risk stratification and prognosis evaluation in the geriatric surgical population.

## Additional files

Additional file 1: Search strategy for PubMed. (DOCX 13 kb)

Additional file 2: Age characteristics. (DOCX 15 kb)

Additional file 3: The association of age and outcomes of interest. (DOCX 14 kb)

Additional file 4: Adjustment for possible confounders. (DOCX 14 kb)

## Abbreviations

CI: Cognitive impairment
CI: Confidence interval
GRADE: Grading of Recommendations, Assessment, Development, and Evaluation
MMSE: Mini-Mental State Examination
NCI: non- Cognitive impairment
NOS: Newcastle Ottawa Quality Assessment Scale
OR: Odds ratio
POD: Postoperative delirium
RCT: Randomized controlled trial
RD: Risk difference
RR: Risk ratio
SMD: Standard mean difference
