# Supplementary material for: Effects of an abnormal mini-mental state examination score on postoperative outcomes in geriatric surgical patients: a meta-analysis
Source: BMC Anesthesiol. 2019 May 15;19:74. doi: 10.1186/s12871-019-0735-5 (PMC6521510; doi:10.1186/s12871-019-0735-5)
Supplement: Supplementary file 1 — Search strategy for PubMed. (DOCX 13 kb) [file 12871_2019_735_MOESM1_ESM.docx]

Search strategy for PubMed

(((((((((((((("Cognition"[Mesh]) OR cognitive[Title/Abstract]) OR ("Delirium"[Mesh]) OR delirium[Title/Abstract]) OR function[Title/Abstract]) OR functional[Title/Abstract]) OR ability[Title/Abstract]) OR quality[Title/Abstract]) OR complication[Title/Abstract]) OR complications[Title/Abstract]) OR outcome[Title/Abstract]) OR outcomes[Title/Abstract]) OR ("Hospitalization"[Mesh]) OR hospitalization[Title/Abstract] OR ("Length of Stay"[Mesh]) OR length of stay[Title/Abstract] OR hospital stay[Title/Abstract] OR length[Title/Abstract]) AND (((((((((((("Surgical Procedures, Operative"[Mesh]) OR surgery[Title/Abstract]) OR surgeries[Title/Abstract]) OR operation[Title/Abstract]) OR operations[Title/Abstract]) OR operative[Title/Abstract]) OR postoperative[Title/Abstract]) OR preoperative[Title/Abstract]) OR pre-operative[Title/Abstract]) OR post-operative[Title/Abstract]) OR hip[Title/Abstract]) OR surgical[Title/Abstract]) OR procedure[Title/Abstract] OR procedures[Title/Abstract]) AND ((((((Mini Mental State Examination[Title/Abstract]) OR MMSE[Title/Abstract]) OR MMSE, Mini Mental State Examination[Title/Abstract]) OR Mini Mental Status Examination[Title/Abstract]) OR MMSE, Mini Mental Status Examination[Title/Abstract]) OR Folstein Mini-Mental State Examination[Title/Abstract]))
