# Supplementary material for: Effects of an abnormal mini-mental state examination score on postoperative outcomes in geriatric surgical patients: a meta-analysis
Source: BMC Anesthesiol. 2019 May 15;19:74. doi: 10.1186/s12871-019-0735-5 (PMC6521510; doi:10.1186/s12871-019-0735-5)
Supplement: Supplementary file 2 — Age characteristics. (DOCX 15 kb) [file 12871_2019_735_MOESM2_ESM.docx]

| **Additional Material 2.** [**Age**](javascript:void(0);) [**characteristics**](javascript:void(0);)**. CI: cognitive impairment, NCI: non-CI, NS: not significant.** | | | | |
| --- | --- | --- | --- | --- |
| **Study** | **age** | **CI** | **NCI** | **P value** |
| Guo 2014 | Age≥75y | 46.5% | 37.3% | NS |
| Häkkinen 2007 | Age，mean(SD） | 83(5) | 77(6) | ＜0.001 |
| Huusko 2000 | Age，mean(range） | 80(67-92) | 80(66-97) | NS |
| Jones 2017 | Age，mean(SD） | 80.3(7.5) | 83.9(7.0) | ＜0.001 |
| Karni 2013 | Age，mean(SD） | 84(5.88) | 82.07(7.09) | NS |
| Lee 2016 | Age，mean(SD） | 72.88(6.20) | 69.9(4.53) | 0.005 |
| Moncada 2005 | Age，mean | 78 | 79 | NS |
| Morghen 2011 | Age，mean(SD） | 84.5(5.8) | 79.2(6.6) | ＜0.001 |
| Otano 2015 | Age，mean(SD） | 86.6(5.7) | 83.5(6.3) | ＜0.001 |
| Rolland 2004 | Age，mean(SD） | 85.9(7.2) | 77.6(7.4) | 0.001 |
| Schaller2012 | Age，mean(SD） | 85.9(6.2) | 82.2(7.2) | ＜0.001 |
