# Supplementary material for: Effects of an abnormal mini-mental state examination score on postoperative outcomes in geriatric surgical patients: a meta-analysis
Source: BMC Anesthesiol. 2019 May 15;19:74. doi: 10.1186/s12871-019-0735-5 (PMC6521510; doi:10.1186/s12871-019-0735-5)
Supplement: Supplementary file 3 — The association of age and outcomes of interest. (DOCX 14 kb) [file 12871_2019_735_MOESM3_ESM.docx]

| **Additional Material 3. The association of age and outcomes of interest. POD: postoperative delirium, NS: not significant.** | | | |
| --- | --- | --- | --- |
| **Study** | **outcomes of interest** | **Age** | **P value** |
| Beloosesky 2002 | In-hospital mortality | Not mentioned | NS |
| Bliemel 2015 | 1-year mortality | 83(9)vs81(8) | 0.002 |
| Brouquet 2010 | POD | 82.7(4.6)vs80.8(4.9) | NS |
| Kalisvaart 2006 | POD | Not mentioned | ＜0.001 |
| Kratz 2015 | POD | 80(6.5)vs76(5.4) | ＜0.001 |
| Osse 2012 | POD | 75.1(3.1)vs76.7(3.9) | NS |
| Reissmüller 2006 | POD | 72.9(5.4)vs71.2(6.2) | NS |
| Ruggiero 2016 | 1-year mortality | 82.47(7.1)vs86.83(7.13) | ＜0.001 |
| Schaller2012 | 1-year mortality | Not mentioned | NS |
| Witlox 2009 | POD | 84.7(5.1)vs82.4(4.6) | 0.04 |
| Yukako 2016 | POD | 80.6(3.8)vs80.0(4.2) | NS |
