# Supplementary material for: Effects of an abnormal mini-mental state examination score on postoperative outcomes in geriatric surgical patients: a meta-analysis
Source: BMC Anesthesiol. 2019 May 15;19:74. doi: 10.1186/s12871-019-0735-5 (PMC6521510; doi:10.1186/s12871-019-0735-5)
Supplement: Supplementary file 4 — Adjustment for possible confounders. (DOCX 14 kb) [file 12871_2019_735_MOESM4_ESM.docx]

| **Additional Material 4. Adjustment for possible confounders.** | | | |
| --- | --- | --- | --- |
| **Study** | **outcomes** | **Confounders** | **Adjusted Effect size** |
| Guo 2014 | 1-year mortality | age, sex | HR 1.65, 95%CI (1.41, 1.78) |
| Kratz 2015 | POD | age, BI, sex, infection | OR 4.18, 95%CI (1.71–10.20) |
| Osse 2012 | POD | age, sex, type of surgery | OR 4.30, 95%CI (1.8–10.69) |
| Reissmüller 2006 | POD | age, alcohol consumption | OR 11.3, 95%CI (2.7–47.7) |
| Schaller2012 | 1-year mortality | age, sex, BMI, comorbidities,  infection, living at home | HR 5.77, 95%CI (1.55, 21.55) |
| Yukako 2016 | POD | age, sex, BMI, operative time，blood loss, tumor stage | OR 7.36, 95%CI (3.19–17.72) |
